# Commentary: Onco-Esthetics Dilemma: Is There a Role for Electrocosmetic-Medical Devices?

**DOI:** 10.3389/fonc.2021.718277

**Published:** 2021-09-30

**Authors:** Andrej Zdravkovic, Lovro Markovic, Richard Crevenna

**Affiliations:** Department of Physical Medicine, Rehabilitation and Occupational Medicine, Medical University of Vienna, Vienna, Austria

**Keywords:** neoplasms, physical and rehabilitation medicine, extracorporeal shockwave therapy, contraindications, recovery of function

## Introduction

With many newly emerging treatment options for most cancer types, as well as treatment efficacy, the number of people living with cancer has been increasing steadily across the globe (1, 2). As a result, the importance of supportive care and rehabilitation has grown substantially (3–6). Palmieri et al. (7) recently authored a review of select therapy modalities, with a focus on the array of cosmetic treatment possibilities in cancer patients, as well as their respective safety. The authors concluded that moderate and periodical use of “cosmetic medical devices”, including massage, are not contraindicated in cancer patients. Although we share the general ethos of the work, we do feel that certain parts of this work require further clarification and the inclusion of the current scientific consensus. We feel that this topic is of great importance to cancer patients and the involved physicians, and could not fail to notice a number of methodological and contentual shortcomings. This commentary will focus primarily on the application of shockwave therapy in cancer patients, as this treatment modality is widely used in the field of physical medicine and rehabilitation, which is the field of expertise of our research group.

## Extracorporeal Shockwave Therapy

The authors elaborate on the use of high energy radial shockwave therapy in cancer patients. The indications included in the article include its use in “kidney stones, [ … ] also in bone, joint, and tendon diseases, and even in cancer.” The use of extracorporeal shockwave therapy (ESWT) originated in the field of urology, where acoustic waves found their application in shockwave lithotripsy, or the dissolution of kidney stones by waves generated by a medical device thenceforth named ‘lithotripter’. Radial shockwave therapy, however, is unlikely to have any effect on kidney stones, as the energy density tends to be the highest on the surface of the skin and the energy density achieved at the needed depth tends to remain far below the domain of high energy focused ESWT (8). As the authors correctly posit, ESWT has a wide range of indications (9, 10). As related to cosmetic treatments, ESWT has been effectively applied in skin ulcers and burn wounds (11), as well as in delayed wound healing (12). In this context, further indications, for which ESWT is commonly used as a second-line or even experimental therapy option include cellulite, lymphedema, palmar and plantar fibromatosis, as well as skin calcinosis (9).

As pertaining to cancer patients, the interest in ESWT has been renewed only recently, after a long period of presumably overcautious indication-setting (13). This change has possibly been brought on by a paradigm shift in the field of physical medicine, in the course of which cancer *per se* was no longer seen as a contraindication for ESWT, but rather a tumor in the treatment area (9). Palmieri et al. (7) cite an *in vitro* study on the effects of ESWT on cancer cells, positing that cell rupture is one of the primary mechanism by which ESWT exerts its effects. Tissue damage has been reported in the literature as a side-effect of high-energy focused ESWT (14). To our knowledge, no such adverse event has been reported after the administration of low-energy focused ESWT, which is the modality much more commonly used.

Although some research has been done on the effects of ESWT on cancer cells, the current consensus does not support its use for this indication (9). Furthermore, in cancer patients with musculoskeletal pain, metastatic disease should be excluded prior to the initiation of ESWT (15–17). A similar recommendation could presumably be made for patients suffering from skin ulcers or delayed wound healing, however, to our knowledge, research on this topic is lacking.

## Possible Misinterpretation of Results of Our Case Report

The authors mention a number of therapy modalities and their application in cancer patients. Of particular interest to us is a possible misinterpretation of the results of a case study by our research group concerning vibration therapy. We reported on a case of application of whole-body vibration therapy in a patient suffering from urinary incontinence after radical prostatectomy (18). Our findings, however, cannot be extrapolated to all cancer patients suffering from urinary incontinence, especially as regarding the safety of the therapy modality.

## Discussion

In the article by Palmieri et al., the effects and safety of select treatment modalities, including but not limited to, the use of medical devices. The article illustrates well the need for further research on topic of supportive care in cancer, while simultaneously presenting some preliminary data on the effects of direct application of said treatment modalities in the area of the tumor. The authors conclude that a “moderate periodical use” of medical devices to this end is supported by the current literature.

Although we share the view that most treatment options should be made available to cancer patients in all stages and that cancer *per se* should not be considered a contraindication for most therapy options, we must stress the need for the inclusion of physicians in decision-making, in order to reduce the risk of adverse events and suboptimal treatment choices. After all, the authors recommend the inclusion of and close observation by dermatologists for patients undergoing aromatherapy. That such a recommendation should not be extended to other, in some cases far more potent therapy modalities, would represent an argument difficult to maintain.

Even though a number of methodological limitations presumably lessen the applicability of reported findings in the clinical setting, we see the published article as a net positive, as it contributes to the field of supportive care in cancer, an area of ever-increasing interest due to the rising number of cancer survivors.

## Author Contributions

AZ and LM analyzed the article. AZ wrote the manuscript. RC supervised the manuscript creation and revised the initial document. All authors contributed to the article and approved the submitted version.

## Funding

Open access publication was funded by the Medical University of Vienna.

## Conflict of Interest

The authors declare that the research was conducted in the absence of any commercial or financial relationships that could be construed as a potential conflict of interest.

## Publisher’s Note

All claims expressed in this article are solely those of the authors and do not necessarily represent those of their affiliated organizations, or those of the publisher, the editors and the reviewers. Any product that may be evaluated in this article, or claim that may be made by its manufacturer, is not guaranteed or endorsed by the publisher.
